# Supplementary material for: A Bayesian network approach incorporating imputation of missing data enables exploratory analysis of complex causal biological relationships
Source: PLoS Genet. 2021 Sep 29;17(9):e1009811. doi: 10.1371/journal.pgen.1009811 (PMC8504979; doi:10.1371/journal.pgen.1009811)
Supplement: S1 Text — (PDF) [file pgen.1009811.s010.pdf]

S1 Text
Computer simulations to evaluate performance

We carried out a number of different simulation studies to evaluate the performance of our proposed approaches.

Data imputation simulation with three variables

While the data imputation methods are aimed towards larger data sets with many variables, it is still useful to investigate their performance in small, simple, networks. A larger network will in effect be composed of many sub network structures and it is of interest to investigate whether the imputation methods for three variables give adequate results without the support of extra reclaimed data that may be acquired from imputing variables elsewhere in a larger network.

Three variable data were simulated under two models. Model 1 was simulated as follows:

$$\begin{aligned}A &\sim \mathcal{N}(10, 1)\\ C &\sim \mathcal{N}(10, 1)\\ B &\sim \mathcal{N}(10 + \beta A + \beta C, 1)\end{aligned}$$

Model 2 was simulated as follows:

$$\begin{aligned}A &\sim \mathcal{N}(10, 1)\\ B &\sim \mathcal{N}(10 + \beta A, 1)\\ C &\sim \mathcal{N}(10 + \beta B, 1)\end{aligned}$$

Here β represents the strength of the relationship between the variables and data were simulated using values $\beta = 0, 0.1, 0.2, 0.3, 0.4$ and 0.5. The data consisted of 2000 individuals and for each data simulation six data sets were created for which a best fit BN was found: (i) The original data with no missing data; (ii) a data set with 1800 individuals each having one missing value (as described below); (iii) a data set with the missing values imputed using our method; (iv) a data set with the missing values imputed using our method with complete training data; (v) a data set with the missing values imputed using the expectation-maximisation (EM) algorithm as implemented in the `bnlearn` R software package [1]; (vi) a data set with the missing values substituted with values taken randomly from non-missing data.

The first set of simulations used data where 1800 individuals had missing values for B and the second set of simulations used data where 1800 individuals had missing values for C. The third set of simulations used data where 600 individuals had missing values for A, 600 individuals had missing values for B and 600 individuals had missing values for C, resulting in 1800 individuals with missing data in total.

Data were simulated 1000 times to evaluate the performance of the different methods. For each model and each value of β, we calculated the proportion of times from the 1000 simulation replicates that the correct model (or an observationally equivalent model) was fitted under each of the six analysis strategies. Specifically, as model 2, "$A \to B \to C$", is equivalent to models "$A \leftarrow B \to C$" and "$A \leftarrow B \leftarrow C$" (since they represent the same dependencies, namely that A and C are independent given B), these were all considered as correct models for model 2. We note that in real data analysis, the ability to distinguish between these models can be achieved through the use of genetic variables that can act as causal anchors [2].

Data imputation simulation with five variables

To demonstrate the benefit of recovering non-missing data in a slightly larger network, we simulated data with five variables. We simulated data for the following model:

$$\begin{aligned} A &\sim \mathcal{N}(10, 1) \\ C &\sim \mathcal{N}(10, 1) \\ E &\sim \mathcal{N}(10, 1) \\ B &\sim \mathcal{N}(10 + \beta A + \beta C, 1) \\ D &\sim \mathcal{N}(10 + \beta C + \beta E, 1) \end{aligned}$$

Again β represents the strength of the relationship between variables, and for each simulated data set β was set to one of $0, 0.1, 0.2, 0.3, 0.4$ and 0.5. Each simulated data set consisted of 2000 individuals and six data sets were created from it: (i) The original data with no missing data; (ii) a data set where either B and D or A and E have missing values (as described below); (iii) a data set with the missing values imputed using our method; (iv) a data set with the missing values imputed using our method with complete training data; (v) a data set with the missing values imputed using the expectation-maximisation (EM) algorithm as implemented in the bnlearn R software package [1]; (vi) a data set with the missing values substituted with values taken randomly from non-missing data. The best fit network was found for each resultant data set and the whole process was repeated for 1000 simulation replicates.

The first set of simulations used data where B and D had missing values such that 600 individuals had missing values for B, 600 individuals had missing values for D and a further 600 individuals had missing values for both B and D. This resulted in a data set where 1800 individuals had at least one variable with missing data and 200 individuals had complete data. The second set of simulations had the same missing data pattern except that A and E were missing instead of B and D.

For each value of β and missing data pattern, the average recall and precision were calculated over the 1000 simulation replicates. Recall (also known as sensitivity) is the proportion of true edges from the simulation model that were actually retrieved in the best fit network, while precision (also called positive predictive value) is the proportion of true edges from the simulation model that appear among the retrieved edges. The recall and precision are calculated for directed edges, where the edges must be in the correct direction. If there are equivalent networks, the best fit network could have directed edges that appear in either direction; these were taken in the direction of the final fitted network (which in our BayesNetty software is always random). As the underlying simulation network has no equivalent networks, this should not occur too often.

We also simulated data for the following simple 5 node model:

$$\begin{aligned} A &\sim \mathcal{N}(10, 1) \\ B &\sim \mathcal{N}(10 + \beta A, 1) \\ C &\sim \mathcal{N}(10 + \beta B, 1) \\ D &\sim \mathcal{N}(10 + \beta C, 1) \\ E &\sim \mathcal{N}(10 + \beta D, 1) \end{aligned}$$

Data were simulated and analysed as described for the previous 5 node model. For this network there are equivalent networks such that any edge could be directed in either direction. Any

network where the nodes are connected in a line (A to E) and no node has more than one parent is equivalent to this network. Therefore in the results the recall and precision correspond to detecting the presence of edges in either direction.

Data imputation simulation with examples from the Bayesian Network Repository

We also simulated data using Bayesian networks from the `bnlearn` Bayesian Network Repository [3] as examples of reference Bayesian networks. Although we are most interested in networks with continuous variables or with mixed continuous/discrete variables, we consider two discrete Bayesian networks, "alarm" and "insurance", previously used by Friedman to compare discrete data imputation methods [4], to demonstrate that our method is also suitable for purely discrete networks. We also consider one continuous network from the repository called "ecoli70". The alarm network has 37 nodes, 46 edges and 509 parameters; the insurance network has 27 nodes, 52 edges and 984 parameters; and the ecoli70 network has 46 nodes, 70 edges and 162 parameters. Data were simulated in R using the `bnlearn` function `rbn` with the BN definitions for each network from the repository. A total of 500 individuals were simulated, with 450 individuals having probabilities of 10%, 20%, 30% or 40% of each variable being set to missing.

For each percentage of missing data, the best fit BN was found for the following six data sets: (i) The original data with no missing data; (ii) a data set with missing values; (iii) a data set with the missing values imputed using our method; (iv) a data set with the missing values imputed using our method with complete training data; (v) a data set with the missing values imputed using the expectation-maximisation (EM) algorithm as implemented in the `bnlearn` R software package [1]; (vi) a data set with the missing values substituted with values taken randomly from non-missing data.

The average recall and precision was calculated for the best fit BN using 100 iterations for each method and percentage of missing data.

Data imputation simulation with many variables

As an example of a more complex model where our data imputation method performs well, we simulated data for a model with 20 SNPs, 10 gene expression variables and one continuous outcome (or "trait") variable as shown in Fig 3 (a). The strengths of the edges were given by parameter β for values $0, 0.1, \cdots 0.5$. SNP variables were simulated using binomial distributions of size 2 with probabilities given by minor allele frequencies ranging from 0.02 to 0.5. For analysis, SNP variables were treated as continuous allele dosages taking values (0, 1, 2), and were constrained to have no parent variables. The expression variables were set to be missing with a probability of 20%. The data consisted of 2000 individuals and so for every simulation replicate there were approximately 215 individuals with complete data. In each simulation, four data sets were created: (i) The original data with no missing data; (ii) a data set with missing values (as described above); (iii) a data set with the missing values imputed using our method; (iv) a data set with the missing values substituted with values taken randomly from non-missing data. The recall and precision were calculated for each value of β and averaged over 100 simulation replicates.

Soft constraint simulations

We started by using a simple simulation model to demonstrate the effect of changing the prior probability of an edge from X to Y when there is indeed an effect from X to Y and X has a parent variable, A, of varying effect, which helps orientate the direction of the edge between X and Y. We simulated data for 250 individuals as follows:

$$\begin{aligned} A &\sim \mathcal{N}(0,1) \\ X &\sim \mathcal{N}(\beta_A A, 1) \\ Y &\sim \mathcal{N}(\beta_X X, 1) \end{aligned}$$

where $\beta_X = 0.5$ and $\beta_A = 0, 0.1, \cdots, 0.5$ and we used 1000 simulation replicates for each value of β_A. We calculated the best fit network assuming the edge A to X is fixed in this direction and fitted separately for different prior probabilities of X to Y $(0, 0.1, \cdots, 0.5)$. The proportion of the best fit models with the edge directed from X to Y was calculated from the 1000 simulation replicates.

The next simulation model was the same except that the edge between X and Y was directed from Y to X. The simulation model for 250 individuals in this case was as follows:

$$\begin{aligned} A &\sim \mathcal{N}(0,1) \\ Y &\sim \mathcal{N}(0,1) \\ X &\sim \mathcal{N}(\beta_A A + \beta_Y Y, 1) \end{aligned}$$

where $\beta_Y = 0.5$ and $\beta_A = 0, 0.1, \cdots, 0.5$, and we simulated data 1000 times for each value of β_A. As before, the best fit model was used to calculate the proportion of times there was an edge from X to Y in the 1000 simulation replicates.

The next simulation investigated the effects of detecting two edges in the correct direction when the prior edge probabilities were varied. The simulation model was as follows:

$$\begin{aligned} A &\sim \mathcal{N}(0,1) \\ B &\sim \mathcal{N}(0,1) \\ X &\sim \mathcal{N}(aA + \beta_B B, 1) \\ Y &\sim \mathcal{N}(\beta_X X, 1) \end{aligned}$$

where $\beta_X = 0.3$ and the edge from A to X was fixed at $a = 0.1, 0.3$ or 0.5. The prior probabilities of B to X were set to $0, 0.05, \cdots, 1$ against every prior probability of X to Y of $0, 0.05, \cdots, 1$. As before, the best fit model was used to calculate the proportion of times there was an edge from X to Y in the 1000 simulation replicates, and also from B to X.

The final simulation demonstrates the effects of varying the prior probabilities of some edges on the ability to detect other edges in best fit models with no prior probabilities. The simulation model shown in Fig 5 (a) was used to simulate data for 500 individuals and the best fit model was found. This was repeated for 10,000 simulated data sets and the proportion of times each edge in the original simulation network appeared in the best fit network was calculated, as well as the average recall and precision. The red edges shown in Fig 5 (a) (labelled with p) had prior probabilities set to $0, 0.1, \cdots, 0.9, 1$. Variables were simulated to have normal distributions with variance 1 and the edges were given by coefficients $\beta = 0.3$ for red, blue and cyan edges (labelled with p, + and x respectively), and $\beta = 0.05$ for all other edges.

Results from soft constraint simulations

Our first simulation demonstrated the effect of changing the prior probability, p, of an edge from X to Y (which automatically sets the prior probability of an edge from Y to X to 1-p) when there is indeed (i) an effect from X to Y or (ii) an effect from Y to X, and X has a parent variable, A, of varying effect, which helps orientate the direction of the edge between X and Y. Since, in this simple example, A is independent of any confounders of X and Y, and there is no direct path from A to Y other than through X, if β_A is non-zero then A can be considered as an instrumental variable or "instrument" for X [5]. S3 Fig shows the proportion of times the best fit BN correctly includes the directed edge from X to Y for various strengths (β_A) of the edge from A to X. Provided β_A is non-zero, increasing the prior probability of an edge from X to Y increases the power for its detection when it exists (S3 Fig (a)), while only slightly increasing the chance of a false positive detection (S3 Fig (b)) when the directed edge is really from Y to X. The larger the value of β_A, the greater the power increase and the less the chance of a false positive detection. When $\beta_A = 0$, so there is no edge from A to X, the proportion of times that edge X to Y is in the best fit model is symmetrical about prior probability 0.5 and follows an identical pattern in both S3 Fig (a) and S3 Fig (b) i.e. is not influenced by the true direction of the causal relationship between X and Y.

The results from our second simulation, investigating varying the prior probabilities for two edges within a 4-variable network, are described in the S4 Fig legend. The results from our final simulation are shown in Fig 5 and are described in the main manuscript text.

References

[1] Scutari, M. (2020). Bayesian network models for incomplete and dynamic data. Statistica Neerlandica pp. 1–23.

[2] Howey, R., Shin, S.-Y., Relton, C., Davey Smith, G., and Cordell, H. J. (2020). Bayesian network analysis incorporating genetic anchors complements conventional Mendelian randomization approaches for exploratory analysis of causal relationships in complex data. PLOS Genetics *16*, e1008198.

[3] Scutari, M. and Denis, J.-B. (2014). Bayesian Networks with Examples in R. (Texts in Statistical Science, Chapman & Hall/CRC (US)).

[4] Friedman, N. (1997). Learning belief networks in the presence of missing values and hidden variables. Proceedings of the fourteenth international conference on machine learning (ICML97) *97*, 125–133.

[5] Pearl, J. and Mackenzie, D. (2018). The Book of Why. (Penguin Books).
