# Supplementary material for: A Bayesian network approach incorporating imputation of missing data enables exploratory analysis of complex causal biological relationships
Source: PLoS Genet. 2021 Sep 29;17(9):e1009811. doi: 10.1371/journal.pgen.1009811 (PMC8504979; doi:10.1371/journal.pgen.1009811)
Supplement: S2 Text — (PDF) [file pgen.1009811.s011.pdf]

S2 Text
Computational considerations and timings of BayesNetty, including use of the Open Message Passing Interface (MPI) for parallel processing

Searching through network space can be very computationally expensive since, at each step of the search algorithm, the network score needs to be evaluated for many potential networks. The network score requires linear regression for each continuous node based upon which parent nodes it has, and for a large network this can involve a lot of calculations which may take some time. As the network score is the sum of each node score, one way to speed up the calculation is to cache the score for each node with each different set of parent nodes. However, this approach may be problematic if the cache becomes too large, which is quite possible for large networks. In BayesNetty a similar idea is used to speed up the computation of the network search. Instead of caching node scores, the network score *difference* of adding, deleting or reversing an edge is recorded. That is, if an edge is modified how does the network score differ, which may be an increase or decrease. At each step of the search, after an edge is modified, the cache of network score differences is updated. The number of network scores needed to be calculated at each step is proportional to the number of nodes in the network. Effectively, when the network is updated, only nodes affected by this update need further calculations to update the cache and the parts of the network which are unaffected (which will be most of it for a large network) will not need to be updated. This approach speeds up calculations considerably and makes it feasible to search networks with hundreds or even thousands of nodes.

Open Message Passing Interface (MPI) has also been implemented in BayesNetty to offer parallel processing as well as the standard serial version. The Open MPI version only uses parallel processing for the search algorithm as this is the most computationally expensive task. In the search algorithm, at each step the different edge updates are calculated in parallel, and so it is not advantageous to have more processors than the number of nodes in the network. Open MPI has extra computational overhead compared to a standard serial program as all of the processors must be managed and communicate with one another at each step, and so the optimum number of processors may be lower than the number of nodes; for small networks it may be quicker not to use Open MPI.

Data were simulated as in Figure 3 (a) using 2000 individuals to compare timings of fitting a network with the following settings: serial (not parallel), Open MPI with 1, 8, 16, 24 and 32 processors. Simulations were performed for larger networks by adding extra nodes replicating the network structure of the original network, giving networks with 31, 62, 124, 248 and 496 nodes. The median time of five different runs was recorded. Varying the number of individuals was also considered, with the number of nodes set to 31.

Timings were also performed for calculating the average network (with 1000 bootstraps) and data imputation for the network with 31 nodes. Missing data was added for data imputation as described previously for this network giving rise to 1796 individuals with missing data. Also, to compare imputation time with that of the `bnlearn` R package EM algorithm, SNPs were removed as the EM algorithm does not handle mixed data. Timings for networks with 11, 22, 44 and 88 nodes were then compared.

Parallel processing to calculate average networks and data imputation can be performed much

more quickly by running multiple instances of the serial version of BayesNetty, and then combining results to get the final result. To calculate the average network, the data is bootstrapped many times and the best fit network fitted; the resultant networks are then combined to get the average network. With enough processors, these bootstrap iterations can all be done in parallel. Similarly, for data imputation, as many processors as individuals with missing data can be used. To investigate the resulting timings, the data imputation and average network were calculated using 1000 processors running the serial version of BayesNetty. The processes were run on a high performance computer with 2 Intel Xeon E5-2699 v4 processors (2.2 GHz), 2.9 GB memory per core.

The timings of BayesNetty to fit a best fit BN are given in the Additional Tables below. The time to fit a BN with 31 nodes, which could be considered reasonably large, using the non-parallel version of BayesNetty takes less than one second. For larger networks the timing is also reasonable taking just over 6 minutes for a network with 496 nodes. Earlier implementations which naively recomputed the network score for each step took over 6 minutes for a network of size 31, over an hour for 62 nodes and was not feasible for 496 nodes. The times can be further decreased with the use of the parallel version of BayesNetty which uses the Open MPI parallel programming system. There is some computational cost using Open MPI due to setup and processors communicating with one another. The setup cost is shown by comparing the computing timings in the MPI(1) column with the serial timings. The parallel timings are much quicker and are around 10 times quicker for the largest networks, although for the network with 31 nodes the parallel timings are actually a bit slower due to the extra cost involved. There is little to be gained from using more than 16 processors and may even be slower for very large networks. The optimum number of processors in general depends on the data structure, data size and the computing system, so some experimentation may be needed.

The timings to calculate an average network are also shown in the Additional Tables below, which shows that by far the quickest approach is to use the simple parallel approach which runs 1000 BayesNetty serial programs and collates the results. Using the parallel version of BayesNetty does speed up the computation by increasing the network search at each step.

The timings to impute data with BayesNetty are also shown below and show a similar pattern to the average network timings for the same reasons. Again it can be seen that it is not advantageous to use more than 16 processors. The imputation timings for the EM algorithm are much quicker than BayesNetty when comparing with the serial version, but, as imputation in BayesNetty can be ran in parallel, it is possible to impute data quicker in BayesNetty.

For most data applications, which typically consist of less than 50 nodes, there is probably no advantage in using the parallel version of BayesNetty to fit a best fit BN. For calculating an average network or imputing data, use of the simple "serial" parallel approach is recommended for larger networks. The BayesNetty website provides scripts to help do this. The timings for larger networks if one uses this approach would be about the same time as it takes to fit one BN in serial, assuming there are enough separate processors.

Best Fit Network Search Timings in Seconds

Nodes	Serial (RT)	MPI(1)	MPI(8)	MPI(16)	MPI(24)	MPI(32)
31	<1	1	1	1	1	1
62	2	3	1	1	1	1
124	13	16	2	2	1	1
248	57	75	13	8	8	7
496	370	402	65	36	39	39

Best Fit Network Search Timings in Seconds

Individuals	Serial (RT)	MPI(1)	MPI(8)	MPI(16)	MPI(24)	MPI(32)
2000	<1	1	1	1	1	1
4000	1	1	1	1	1	1
8000	3	3	2	1	1	1
16000	9	8	3	2	2	2
32000	25	24	7	4	4	4
64000	41	36	13	10	7	7
128000	91	73	23	18	15	14

Average Network Timings in Seconds

Nodes	Serial (RT)	Parallel 1000 (RT)	MPI(1)	MPI(8)	MPI(16)	MPI(24)	MPI(32)
31	732	5	870	205	145	140	132

Data Imputation Timings in Seconds

Nodes	Serial (RT)	Parallel 1000 (RT)	MPI(1)	MPI(8)	MPI(16)	MPI(24)	MPI(32)
31	631	4	965	223	169	184	232

Data Imputation Timings in Seconds

Nodes	Serial (RT)	Parallel 1000 (RT)	Serial (CT)	Parallel 1000 (CT)	EM
11	98	3	88	3	12
22	451	5	626	5	22
33	1770	8	697	6	36
44	9096	29	4641	8	76

Additional Tables for Text S2: Time in seconds to perform various calculations with the BayesNetty software. The standard non-parallel times are given by the serial column. Parallel processor times using Open MPI are denoted by MPI and the number of processors in brackets (note MPI(1) uses Open MPI but is not in parallel). The Parallel 1000 column refers to 1000 BayesNetty serial programs running in parallel to calculate either the average network or data imputation, with the results collated to give the final answer. Imputation with random training is denoted by RT and for complete training by CT.
