# Supplementary material for: A Bayesian network approach incorporating imputation of missing data enables exploratory analysis of complex causal biological relationships
Source: PLoS Genet. 2021 Sep 29;17(9):e1009811. doi: 10.1371/journal.pgen.1009811 (PMC8504979; doi:10.1371/journal.pgen.1009811)
Supplement: S3 Text — (PDF) [file pgen.1009811.s012.pdf]

S3 Text
Exploration of the effect of varying the subset percentage size taken in step 1 of the BayesNetty imputation algorithm

Here we explore how the performance of our imputation algorithm varies according to the size of the data subset taken at step 1, when imputing an individual with missing data. To show any effect, it was necessary to use an example where there is a reasonable difference in recall and precision between using imputation and simply replacing the missing data with randomly selected data. (Since, if the recall and precision is impacted by the quality of imputation, then we would expect to have poorer recall and precision for poorer quality imputed data). To do this we use the "ecoli70" network from the `bnlearn` Bayesian Network Repository [1]. The "ecoli70" network has 46 nodes, 70 edges and 162 parameters. Data were simulated in R using the `bnlearn` function `rbn` with the BN definition from the repository. Data were simulated for 500 individuals, 450 of which had probabilities of 10%, 20%, 30% or 40% of each value being set to missing. We varied the subset percentage size used within step 1 of the imputation algorithm by 10% increments between 10% and 100%. Data were simulated 1000 times for each missing data percentage and each subset percentage, and the average recall and precision calculated.

S8 Fig shows the recall and precision of BayesNetty using the two different versions of the imputation algorithm i.e. with random training data (RT) and complete training data (CT). As perhaps expected, it is generally seen that using a larger percentage of the data results in higher recall and precision. In particular, when using the complete training data and when the subset percentage is low, the recall and precision is also low. As there are only 50 individuals with complete data, the complete training data imputation is affected more than the random training data imputation which uses a potentially much larger number of individuals.

Closer inspection of the results shows that the recall and precision may not always increase when the subset percentage is increased. For imputation with complete training data and higher percentages of missing data, the recall and precision levels off, and in some cases even decreases slightly. For imputation with random training data, the recall and precision tends to increase earlier before levelling off. The extra variation that replacing the missing values with randomly selected data provides may explain why the recall and precision tends to increase when the subset percentage is increased.

We believe that our choice of 90% for the subset percentage size is reasonable, as it uses much of the available data while still incorporating network uncertainty. This is particularly important for imputation with complete training data, where the only source of variation in the training data used (and thus in the fitted network) is the different subset taken at each invocation of imputation step 1.

References

[1] Scutari, M. and Denis, J.-B. (2014). Bayesian Networks with Examples in R. (Texts in Statistical Science, Chapman & Hall/CRC (US)).
