# Supplementary material for: A Bayesian network approach incorporating imputation of missing data enables exploratory analysis of complex causal biological relationships
Source: PLoS Genet. 2021 Sep 29;17(9):e1009811. doi: 10.1371/journal.pgen.1009811 (PMC8504979; doi:10.1371/journal.pgen.1009811)
Supplement: Attachment — Submitted filename: ResponseToReviewers_BayesNettyPaper_June6th.docx [file pgen.1009811.s013.docx]

*We thank the editors and reviewers for their helpful suggestions which have considerably improved the manuscript. Please see our list of point-by-point responses below.*

Dear Heather,

Thank you very much for submitting your Research Article entitled 'A Bayesian network approach incorporating imputation of missing data enables exploratory analysis of complex causal biological relationships' to PLOS Genetics.

You can find below the comments from three reviewers, recommending major revisions. While the paper has been well received, all reviewers agree that the methodological contributions should be supported by more comprehensive literature review and by a more detailed discussion of the underlying assumptions. They also ask for some clarifications on the experimental evaluation.

Should you decide to revise the manuscript for further consideration here, your revisions should address the specific points made by each reviewer. We will also require a detailed list of your responses to the review comments and a description of the changes you have made in the manuscript.

If you decide to revise the manuscript for further consideration at PLOS Genetics, please aim to resubmit within the next 60 days, unless it will take extra time to address the concerns of the reviewers, in which case we would appreciate an expected resubmission date by email to plosgenetics@plos.org.

If present, accompanying reviewer attachments are included with this email; please notify the journal office if any appear to be missing. They will also be available for download from the link below. You can use this link to log into the system when you are ready to submit a revised version, having first consulted our [Submission Checklist](https://eur03.safelinks.protection.outlook.com/?url=http%3A%2F%2Fjournals.plos.org%2Fplosgenetics%2Fs%2Fsubmit-now%23loc-submission-checklist&data=04%7C01%7Cheather.cordell%40newcastle.ac.uk%7Cee828810aa184543ea0908d8e8be5bf6%7C9c5012c9b61644c2a91766814fbe3e87%7C1%7C0%7C637515252610238242%7CUnknown%7CTWFpbGZsb3d8eyJWIjoiMC4wLjAwMDAiLCJQIjoiV2luMzIiLCJBTiI6Ik1haWwiLCJXVCI6Mn0%3D%7C3000&sdata=7mpAALbBu20Ts1HD9pYFq89xqcaEo%2B7HDXdzdDB1iJ8%3D&reserved=0).

To enhance the reproducibility of your results, we recommend that you deposit your laboratory protocols in protocols.io, where a protocol can be assigned its own identifier (DOI) such that it can be cited independently in the future. For instructions see our [guidelines](https://eur03.safelinks.protection.outlook.com/?url=http%3A%2F%2Fjournals.plos.org%2Fplosgenetics%2Fs%2Fsubmission-guidelines%23loc-materials-and-methods&data=04%7C01%7Cheather.cordell%40newcastle.ac.uk%7Cee828810aa184543ea0908d8e8be5bf6%7C9c5012c9b61644c2a91766814fbe3e87%7C1%7C0%7C637515252610248203%7CUnknown%7CTWFpbGZsb3d8eyJWIjoiMC4wLjAwMDAiLCJQIjoiV2luMzIiLCJBTiI6Ik1haWwiLCJXVCI6Mn0%3D%7C3000&sdata=SJZr9ddKmlz%2BCovDHNafSr8BT%2Frh%2BKwkxdH0alolnJk%3D&reserved=0).

Please be aware that our [data availability policy](https://eur03.safelinks.protection.outlook.com/?url=http%3A%2F%2Fjournals.plos.org%2Fplosgenetics%2Fs%2Fdata-availability&data=04%7C01%7Cheather.cordell%40newcastle.ac.uk%7Cee828810aa184543ea0908d8e8be5bf6%7C9c5012c9b61644c2a91766814fbe3e87%7C1%7C0%7C637515252610248203%7CUnknown%7CTWFpbGZsb3d8eyJWIjoiMC4wLjAwMDAiLCJQIjoiV2luMzIiLCJBTiI6Ik1haWwiLCJXVCI6Mn0%3D%7C3000&sdata=atoj8D63qjXoVk7cplCQlDKMTcYCDeBM3sg1jD3Y0dc%3D&reserved=0) requires that all numerical data underlying graphs or summary statistics are included with the submission, and you will need to provide this upon resubmission if not already present. In addition, we do not permit the inclusion of phrases such as "data not shown" or "unpublished results" in manuscripts. All points should be backed up by data provided with the submission.

While revising your submission, please upload your figure files to the [Preflight Analysis and Conversion Engine](https://eur03.safelinks.protection.outlook.com/?url=http%3A%2F%2Fpace.apexcovantage.com%2F&data=04%7C01%7Cheather.cordell%40newcastle.ac.uk%7Cee828810aa184543ea0908d8e8be5bf6%7C9c5012c9b61644c2a91766814fbe3e87%7C1%7C0%7C637515252610248203%7CUnknown%7CTWFpbGZsb3d8eyJWIjoiMC4wLjAwMDAiLCJQIjoiV2luMzIiLCJBTiI6Ik1haWwiLCJXVCI6Mn0%3D%7C3000&sdata=glodlqhCPOBR6xKzKoA7V1e0%2BgKNATGXhx6uvdoF39s%3D&reserved=0) (PACE) digital diagnostic tool.  PACE helps ensure that figures meet PLOS requirements. To use PACE, you must first register as a user. Then, login and navigate to the UPLOAD tab, where you will find detailed instructions on how to use the tool. If you encounter any issues or have any questions when using PACE, please email us at figures@plos.org.

PLOS has incorporated [Similarity Check](https://eur03.safelinks.protection.outlook.com/?url=http%3A%2F%2Fwww.crossref.org%2Fcrosscheck.html&data=04%7C01%7Cheather.cordell%40newcastle.ac.uk%7Cee828810aa184543ea0908d8e8be5bf6%7C9c5012c9b61644c2a91766814fbe3e87%7C1%7C0%7C637515252610258155%7CUnknown%7CTWFpbGZsb3d8eyJWIjoiMC4wLjAwMDAiLCJQIjoiV2luMzIiLCJBTiI6Ik1haWwiLCJXVCI6Mn0%3D%7C3000&sdata=6fAk3CdvMgKorgExCaLwCUnMxVGmTAJhIFDmvzGNMFk%3D&reserved=0), powered by iThenticate, into its journal-wide submission system in order to screen submitted content for originality before publication. Each PLOS journal undertakes screening on a proportion of submitted articles. You will be contacted if needed following the screening process.

To resubmit, use the link below and 'Revise Submission' in the 'Submissions Needing Revision' folder.


We are sorry that we cannot be more positive about your manuscript at this stage. Please do not hesitate to contact us if you have any concerns or questions.

Yours sincerely,

Marco Scutari
Guest Editor
PLOS Genetics

David Balding
Section Editor: Methods
PLOS Genetics

Reviewer's Responses to Questions

**Comments to the Authors:
Please note here if the review is uploaded as an attachment.**

Reviewer #1: This is pretty good stuff. It’s very thorough. Very well-written

*We thank the reviewer for their comment.*

I have a couple major points:

Causality (or lack thereof in BN semantics): The authors should address the fact that edges in BNs aren’t causal and only represent statistical dependence (or, rather, conditional independence) relationships. There’s no expectation that the directions of arrows in a BN really mean that much, since, under many conditions, there’s no statistical difference. Consider the paragraph in lines 12-21. Nothing about BN semantics implies causality. The authors should either talk about under which conditions causality can be inferred (such as in Heckerman, https://arxiv.org/abs/1302.4958 ), or drop the references to causality (Or cite some of the causal-BN proponents in a balanced discussion).

*We apologise for not covering this aspect previously. We had discussed this aspect in some detail in our previous paper (Howey et al. 2020: https://pubmed.ncbi.nlm.nih.gov/32119656/) and so we had not considered it necessary to repeat the discussion in the current manuscript. However, as requested, we have now included an even more detailed discussion (!) of this issue within the Introduction of the current manuscript.*

The authors should also consider if edges they’re trying to recreate in networks are edges that are constrained to point in one direction or if they’re edges that could have been in the other direction. This has implications for how their edge-recreation algorithms are scored.

*Whether or not the edges were constrained to point in a particular direction at the analysis stage depends on what constraints were implemented within each simulation experiment (or real data analysis). If constraints were applied, then any network violating the constraints was not considered (i.e. effectively scored minus infinity). When evaluating the algorithms through calculating the precision/recall based on comparing output networks to the underlying simulation model, networks that could not be distinguished (i.e. were observationally equivalent and therefore achieved the same score) were all considered equally correct, and thus any edges in these networks whose direction could not be distinguished were counted as correct for the purposes of calculating recall/precision, regardless of the direction in which they were identified.*

*We had previously attempted to explain this in the manuscript. For example, in relation to the first simulation experiment, we had we already stated (see^d^ 2nd paragraph of Results section): “Note that equivalent networks, representing the same dependencies/independencies between variables, cannot be distinguished and are all considered as “correct”. This was elaborated in more detail in Supplementary Text S1 in the section “Data imputation simulation with three variables”, where we stated: “As model 2, “A → B → C”, is equivalent to models “A ← B → C” and “A ← B ← C” (since they represent the same dependencies, namely that A and C are independent given B), these were all considered as correct models for model 2”.*

*Similarly, in the S1 Text section “Data imputation simulation with five variables” we had previously stated (in relation to model 1): “The recall and precision are calculated for directed edges, where the edges must be in the correct direction. If there are equivalent networks, the best fit network could have directed edges that appear in either direction; these were taken in the direction of the final fitted network (which in our BayesNetty software is always random). As the underlying simulation network has no equivalent networks, this should not occur too often”.*

*Similarly, in the S1 Text section “Data imputation simulation with five variables” we had previously stated (in relation to model 2): “For this network there are equivalent networks such that any edge could be directed in either direction. Any network where the nodes are connected in a line (A to E) and no node has more than one parent is equivalent to this network. Therefore in the results the recall and precision correspond to detecting the presence of edges in either direction”.*

*In general, within our software package BayesNetty, an edge is constrained to point in a particular direction when the node from which it emanates is a genetic variable. We had already mentioned this in Text S1 in the section “Data imputation simulation with three variables” where we had stated: “We note that in real data analysis, the ability to distinguish between these models can be achieved through the use of genetic variables that can act as causal anchors [2]” and, in the section “Data imputation simulation with many variables”, where we had stated: “For analysis, SNP variables were constrained to have no parent variables”.*

*We have now added some further clarification in several places within the main text regarding this issue. In particular, in the Introduction section we have added the following paragraph:*

*“An advantage of using genetic data in this type of analysis is the fact that genes are assigned at birth, and so can act as ``causal anchors'', whereby arrows (interpreted as causal relationships) should only be directed outwards from genetic variables towards the non-genetic variables that they influence. This constraint provides a practical way to choose between BNs that are observationally equivalent; if an acausal BN is supposed to reflect the independencies implied by the corresponding causal BN, then it makes sense to only consider BNs that obey the desired constraint. Our software package, BayesNetty, specifically enforces this constraint by only considering BNs that have arrows coming out from (rather than going into) any variables that have been labelled as genetic variables. In addition, it is possible within BayesNetty to ``blacklist'' certain arrows such that edges between specified nodes are not allowed to exist, effectively constraining the direction that any arrow between these nodes can take.”*

*And, in the Discussion section, we say:*

*“We also considered an extension to our BN approach in the form of soft constraints, whereby a prior probability, p, can be assigned to any directed edge with 1-p automatically assigned to the reverse direction. This is in addition to incorporating hard constraints in the form of “white" directed edges which must be included in any BN and “black" directed edges which must be excluded. (If the white list contains both directions between two nodes, then the edge must be included but may be in any direction).”*

Why throw out whole samples?: I’m not sure, but I assume that when learning BNs there’s no need to throw out a sample simply because it has a few missing values. Since BNs are built using local conditional probability tables, each local dependency computation can be computed with as much data as is available for the variables in question. So a sample can easily contribute to the CPTs for which it contains values, while being skipped for the computations involving the values it is missing. It would seem this is the natural baseline for comparison of imputation methods.

*This is an attractive idea, but, in fact, it is not possible to use different numbers of observations (=people) when modelling the different local dependencies, as the resulting log likelihood (or score based on the negative AIC or negative BIC) would end up being based on different effective sample sizes (=numbers of observations) for different networks. This would mean that we would not be able to directly compare the network scores for different networks in order to choose the best fitting network(s), as we would not be comparing “like with like” in terms of the amount of information available.*

There’s a lot of data double dipping, and I suspect this leads to inflated results. The whole scheme for estimating precision and recall seems to have been created de novo, since no citations are provided. In the description of this method, the same imputation method is used in step 1 and in step 5(ii). Since the methods tested in step 5 are compared to the results of step 1, one assumes that method (ii) should do the best by this metric since it’s the same method that was used to generate the data. This is what is played out in the results.

*We disagree that there is any “double dipping” in relation to step 1 and step 5(ii) of the standard algorithm for estimating precision and recall. The network from step 1 is effectively being used as the basis of a new simulation study (with a single simulation replicate, generating the data described in step 4). Within the simulation study, the step 1 network is indeed the `true’ network, while the step 5 networks are the estimated networks obtained when analysing the simulated data that was generated from this true step 1 network. We have added some text to the description of the algorithm to help clarify this point.*

*A question does arise about how representative this step 1 network is of the unknown true network underlying the real data. We had already mentioned this issue in a subsequent paragraph, but we have added a further sentence of description to help clarify this point.*

*The validity of the alternative algorithm, proposed for use in situations where it is not computationally feasible to run many iterations of the original algorithm, will depend on the extent to which the step 3 best fit network for the simulated full data set can be considered an accurate representation of the step 1 network used as the generating model. We have added a sentence at the end of this subsection mentioning this fact.*

A philosophical point:
The problem of recapitulating edges from biological networks is very difficult. It’s also difficult to know if it’s being done properly, since it’s hard to find such networks with large datasets where the true network is known with certainty. Thus, authors usually present toy examples of networks with a handful of nodes and simulated data, then argue that their recapitulation algorithm works on this toy example, so it might therefore work on real data. However, the gulf between a toy network of 5 nodes and actual biology on an omics scale is staggering. It might be interesting to see some kind of samplesize calculation that estimated the number of samples required to do this on a much bigger scale.

*Although we started by focussing on toy examples with 3 or 5 nodes, we did in fact extend our simulations to more complex models (see Fig 3(a) and Fig 5(a)). We have now added in a further more complex simulation model (see the ROC curves described at the end of Results section and in S8 Fig).*

Minor Points:
Introduction:
There’s some confusion at the beginning about whether the point of this is to recapitulate known network’s structure or to use the BN to perform imputation of missing data. Bit of Chicken and Egg issue, really. It’s not clear which of these is the primary task.

*We had already mentioned in the introduction that our imputation method was “designed to handle missing data in order to improve identification of the best fit network (or networks)”. By this, we meant to imply that this was indeed the primary aim. The fact that we also use BNs to perform the actual imputation is a secondary issue. We have added a further sentence of clarification to the Introduction to emphasize this fact:*

*“We note that this is a different goal from trying to obtain the most accurate imputation of missing data; although one might hope that accurate imputation of missing data would result in accurate identification of the best fit network, it is the latter (accurate identification of the best fit network) rather than the former (accurate imputation of missing data) that is our primary goal.”*

*Note that we had also stated in the “Data imputation” subsection of the Methods section: “The purpose of our imputation algorithm is to enable generation of the most accurate final network, which does not necessarily correspond to producing the most accurate estimate of the missing data per se for any particular individual or variable”.*

*In addition, we had also stated in the Abstract “We present a new imputation method designed to increase the power to detect causal relationships”. Again, by this, we meant to imply that the detection of causal relationships was the primary aim (which can - up to a point - be achieved through improved identification of the best fit network (or networks)). We have now changed “causal relationships” to “potential causal relationships” in the Abstract, in light of some of the discussion about causality that has now been added to the Introduction.*

Methods:
Couldn’t imputation be improved by not throwing out “nearby variables” that have 1 or more missing value?

*As stated in the text, nearby variables consist of all the other variables that have connecting edges to the variable that is missing in the index individual, and that also have non-missing data for the index individual. Since we want to use the values of these nearby variables to calculate the distance between the index individual and every other individual (in order to choose the most appropriate nearest neighbour individual), it is not possible for this purpose to use any nearby variable that has missing data.*

Not sure if they justify the substitution of nearest neighbor variables for the case of continuous variables. I guess the idea is to use residuals to get a combination of near variables, but it’s not clear why this is preferable. Does just using the near variables result in poor performance?

*The reviewer seems to be confused in their terminology above between nearest neighbor *individuals* (=people) and nearby *variables* (=variables). Is the reviewer referring here to the modification in which we construct a new variable (which is not in the original data) to be used as a nearby variable?*

*We have now slightly expanded the rationale for this in the text in the “Modifications to the data imputation algorithm” subsection of the Methods:*

*“We then use variable v rather than y as a nearby variable for informing the choice of nearest neighbour to impute the missing value of z, since y depends on both x and z, whereas v depends mostly on z”.*

Interesting to include variable Bayesian priors on network edges based on belief, however I think the problem with this is finding a principled way of assigning priors without biasing your results. If the authors have intuitions about how best to do so, it would be a welcome contribution.

*The rationale for including an option to use variable Bayesian priors on network edges based on belief was the idea that this might improve power in the situation where such belief is well-supported by prior evidence (or scientific theory), without seriously biasing the results if the belief turned out to be misplaced (as the prior would be overcome by the data). This idea seems to be largely borne out by our simulation study. Methods for assigning such priors based on prior evidence or scientific theory are likely to be context/application specific, and so we are not able to provide any particular intuition about this, although we note that there is a literature on expert elicitation of Bayesian priors.*

*We have now added a couple of sentences making the above points to the “Utility of applying soft constraints” subsection of the Results section (lines 227-331).*

The authors are talking about methylation of SNPs, and then say that “100” is a “large BN”. Shouldn’t there be about 400k methylation sites and 6 million SNPs?

*We never intended to suggest that BNs would be capable of modelling 400k methylation sites and 6 million SNPs in a single network! This would certainly be computationally infeasible. The natural first step for this type of analysis is to filter down to a smaller set of variables based on some level of pairwise correlation. Since we were using this data set purely for illustrative purposes, we used the same filtered set of variants as was used by Clark et al. We have now added a sentence to the Methods (lines 713-715) to clarify this point. We have also added the qualifier “to model the candidate triplets” to the heading “Use of a large BN” to emphasize the fact that “large” is simply being considered in comparison to the “small” (6-variable) networks that are considered when considering the triplets individually.*

Results:

There’s no method explanation for how the power plots of Figure 1 were generated. Was it using the Precision and Recall methodology?

*Figure 1 is first mentioned in the Results subsection “Imputation of small networks (3 or 5 variables respectively)”, following a sentence that says “We start by using computer simulations to investigate…” and preceding a sentence that says “(See S1 Text for details of the simulation models”). Later, within the Methods section of the main manuscript, we say “We carried out a number of different simulation studies to evaluate the performance of our proposed approaches. For more details of these, please see S1 Text.”*

*These sentences were supposed to clearly point the reader to S1 Text which is where full details of the method explanation can be found! Within S1 Text, in the section “Data imputation simulation with three variables” (which corresponds to what is being shown in Figure 1), we had stated:*

*“Data were simulated 1000 times to compute powers and type I errors. For each model and each value of β, we calculated the proportion of times from the 1000 simulation*

*replicates that the correct model was fitted under each of the six analysis strategies. As model 2, “A → B → C”, is equivalent to models “A ← B → C” and “A ← B ← C” (since they represent the same dependencies, namely that A and C are independent given B), these were all considered as correct models for model 2.”*

*Note that we have now slightly edited the phrasing in these sentences in response to the comments of another reviewer.*

*Therefore, the powers and type I errors in Figure 1 - now described as the “proportion of best fit networks that correctly identified the network structure used in the simulation*

*model (or an observationally equivalent network)” in response to the comments of another reviewer - were calculated exactly as described above (and as described in S1 Text), and NOT using the Precision and Recall methodology that is described later.*

Figure 2 seems to be based on the Precision Recall methodology that biases toward the authors’ imputation method.

*Figure 2 was calculated using the *****measured***** precision and recall from the computer simulations (rather than the *****estimated***** precision and recall method designed for use with real data described later - which, as mentioned, we do not in any case consider particularly likely to be biased). This was already mentioned in the main text:*

*“Recall (also known as sensitivity) is the proportion of true edges that were actually retrieved in the best fit network, while precision (also called positive predictive value) is the proportion of true edges among the retrieved edges.”*

*We have added a similar sentence to S1Text to further clarify this point:*

*“Recall (also known as sensitivity) is the proportion of true edges from the simulation model that were actually retrieved in the best fit network, while precision (also called positive predictive value) is the proportion of true edges from the simulation model that appear among the retrieved edges.”*

The CIT and BN results might benefit from an AUC analysis where the accuracy across various thresholds can be visualized. This may be aide the discussion about thresholds of 0.8 and of 0.65.

*We have now added in an AUC (ROC curve) analysis, comparing BN and CIT, using computer simulations based on the real early arthritis data. See end of Results section and S8 Fig. As the real early arthritis data is a real data set, the truth (or otherwise) of any identified relationships using this data set is obviously unknown, and it so cannot be directly used to compare true and false detections (as required for an AUC analysis).*

Comparison of CIT and BN results are also a little unsatisfying. As I understand it, the CIT results are from the authors’ prior work, and there’s no expectation that they’re more correct than the BN results. This portion is sort of interesting, and it would probably be remiss of the authors not to make such a comparison if they’re comparing two methods for doing the same thing on the same data; but it could perhaps be summarized or moved to the supplement for the reader interested in comparing these methods.

*This example was supposed to be purely illustrative, but we think it important (particularly for a journal with an applied focus like PLOS Genetics) to show the method at work in practice, in application to real data. We have added a sentence (lines 344-347) pointing out that, as this is a real data set, the truth (or otherwise) of any identified relationships is obviously unknown, and reiterating the fact that further work will be required to confirm (or not) the truth of the potential causal relationships identified by CIT and/or BN.*

Reviewer #2: Review

In this paper the authors present a novel method to account for missing data when building a Bayesian network based on high dimensional genetic data. In this setting, restricting the analysis to individuals with complete data (i.e. no missing values) can result in a dramatic loss of precision and potentially bias. The author’s propose the use of an algorithm that incorporates nearest neighbour imputation to fill in the missing data.

Simulations are performed to assess the performance of the approach, software is provided and the method is nicely demonstrated on an applied example

Comments

Page 8:
Line 67 Some use the term `complete case’ in place of `Full’ which could be mentioned

*We have now mentioned this.*

Line 82: Can you make clear here or earlier on that you only consider DAGs (if that’s the case?)

*We had already noted in the Introduction (line 14) that “A BN comprises a graphical model known as a directed acyclic graph (DAG)”. Therefore, by definition, any method that fits BNs will only consider DAGs.*

Line 94: Can you clarify if the ground truth of the 46, 37 and 27-node network data sets in the BNLearn repository is known?

*The structure and generating parameters of the 46, 37 and 27-node network data sets in the BNLearn repository are indeed known. We have now clarified this in the manuscript.*

Page 9,
Line 120
Presumably priors must always be specified, they are not an optional extra? I think you may discuss this later on in the methods, but I was a bit confused at this point. Can you say something here about how one should incorporate prior information, or set priors if they want them to be informative/non-informative?

*It is not actually necessary to specify priors - the term “Bayesian” within BNs does not imply the use of a Bayesian – as opposed to a frequentist – paradigm, but rather refers to the fact that certain calculations rely heavily on Bayes’ theorem. We have now noted this fact on lines 73-75. There exist both Bayesian and frequentist algorithms for searching for networks that are well supported by the data, as we now note on line 70. As now mentioned on line 107, in our own software package we use the (frequentist) score-based algorithm from the R package bnlearn as a basis. A brief description of the algorithm is given in the “BayesNetty software” subsection of the Methods section. The interested reader can obviously consult the referenced books (Scutari M, Denis JB. Bayesian Networks with Examples in R; Nagarajan R, Scutari M, Lebre S. Bayesian Networks in R: with Applications in Systems Biology) for more details concerning the algorithm.*

Line 123: Is A an instrument for X?

*You could certainly consider a parental variable A for child variable X, that helps orient the direction of the edge between X and Y, as an instrument - although, strictly speaking, a valid instrumental variable needs to satisfy additional conditions - it needs to be independent of any confounders of X and Y, and there needs to be no direct path from A to Y other than through X. In our simple example, these conditions hold, and so A is indeed an instrument for X, as we now note in lines 236-238.*

Line 127

Can you say anything general about when increasing the prior for an edge will effect the chances of correctly/incorrectly detecting an edge between any other two variables? Is it as simple as saying that if there is a path from , say, A to Y in the DAG ? i.e. The pathway is `open’

*It seems reasonable to conclude that the results described in the section “Utility of applying soft constraints” (and in the S4 Fig legend, as now mentioned on line 248), and shown in S3 Fig, S4 Fig and Fig 5, do indeed correspond to seeing an improvement in the chances of detection when the pathway is ‘open’. However we have not, strictly speaking, carried out any formal mathematical proof of this, and so we have refrained from making any such general statement.*

Line 136. You mention that some edges are not assigned prior probabilities at all. Now I’m confused! I would have thought that you would have to assign a prior for a Bayesian method to ?

*As mentioned above, it is not actually necessary to specify priors - the term “Bayesian” within BNs does not imply the use of a Bayesian – as opposed to a frequentist – paradigm, but rather refers to the fact that certain calculations rely heavily on Bayes’ theorem. And, as now mentioned on line 107, in our own software package we use the (frequentist) score-based algorithm from the R package bnlearn as a basis*

Page 10
Line 179 Did you use a multiplicity correction in your work?

*All CIT p-values were nominal i.e. NOT adjusted for multiple testing. We have now added the word “nominal” in several places in the text and in the Fig 6 and Fig 7 legends to emphasize this fact.*

*As mentioned in the Table 1 legend, results for the CIT were reported in Table II of Clark et al. [18] when any nominal p-value was less than 0.05 (but focusing on those triplets also satisfying the CIT permutation false-discovery rate (FDR) threshold of 0.05). We have now added some additional text to the main text (line 309) and to the Table 1 legend to point out that this effectively provides an adjustment for multiple testing.*

Page 14 lines 297-8 I’m slightly confused by the statement `applying a prior probability conditional on the event that it exists’? Can you clarify, this (see earlier comments) and that you are using soft and hard priors

*As mentioned in this section of the Discussion, hard constraints correspond to “white" edges which* ****must***** be included in any BN and “black" edges which must be* ****excluded*****. We have now added the further clarification that these are *****directed**** *edges, and that, if the white list contains both directions between two nodes, then the edge must be included but may be in any direction.*

*Although the option exists within our software to specify “white” and “black” edges, we have not specifically implemented this within the current manuscript, other than in the special case when variables are defined to be genetic variables and so are constrained to only have arrows directed outward (towards the non-genetic variables that they influence).*

*We apologise for the confusion regarding our statement in the Discussion “a prior probability can be assigned to the direction of an edge, conditional on the event that it exists”. Soft constraints simply correspond to setting the prior probability of a directed edge to a particular value, say p, with the opposite direction automatically set to 1-p, as now described in the “Soft Constraints” section of the Methods. We have now amended the statement previously made in the Discussion accordingly.*

Page 15
Line 377. Is this threshold 0.6?

*The threshold is not a set value (such as 0.6), but rather will differ according to the properties of the average best network, as described in the references provided (Scutari M, Denis JB. Bayesian Networks with Examples in R; Scutari M, Nagarajan R. Identifying significant edges in graphical models of molecular networks). We have added the words “(data set dependent)” to emphasize this aspect.*

Page 16
Line 408 Not sure if this definition of MAR is correct. Yes outcome dependent missingness is MNAR. But it is possible that missing values are higher than average (or biased), say, but are correctly imputed using nearest neighbour methods (that is, using observed data)

*We have slightly amended this statement. In general, our method is designed for the situation where one would expect the missingness of a given variable to be dependent only on the values of other measured variables, which corresponds to MAR rather than MNAR. Of course, it is possible that in some circumstances MNAR might still lead to values that are correctly imputed using nearest neighbour methods - but it is unclear under what circumstances this property would be guaranteed.*

Line 436. Is this approach related to the MICE procedure (multiple imputation using chained equations)?

*We have not explored this question in detail, but from a theoretical standpoint, we do not consider our procedure to be closely related to the MICE procedure. In particular, although there is an element of randomness in our algorithm (on account of taking a 90% subset of individuals chosen from all the individuals without replacement, as the first step in imputing the data for an index individual), we do NOT perform multiple imputations of the missing data for any individual, i.e. in our algorithm the data is imputed only once. This contrasts with MICE in which the imputations are repeated multiple times, as the algorithm cycles over the missing data values (https://onlinelibrary.wiley.com/doi/full/10.1002/mpr.329).*

*Within the MICE procedure, a series of regression models are run whereby each variable with missing data is modelled conditional upon the other variables in the data. This does have some similarities with our procedure, in as much as we use other “nearby” variables in the data to determine the nearest neighbour individual who should be used to impute the values of the index person. However, unlike MICE, we do not directly use these nearby variables to impute the value of the missing variable, but rather use them to help choose the nearest neighbour individual.*

*We have added some discussion of this issue to the Discussion section of the manuscript (lines 456-469).*

Reviewer #3: This manuscript describes an adaptation of an existing algorithm for fitting Bayesian networks (BN) when some data are missing in a subset of samples, by using nearest neighbour imputation, where the variables to be used in learning the nearest neighbour are themselves learnt from a BN.

This is a nice idea, and the approach is evaluated through simulation and real data analysis. However, it is not clear which of the two imputation methods are optimal in which situations, when they outperform other baselines, computational requirements, and whether the proposed method actually outperforms CIT or not in the real data analysis - how can this comparison be evaluated without any ground truth knowledge?

*In terms of which of the two imputation methods are optimal in which situations, we had already covered our *expectation* in this regard (and the ensuing rationale for the “imputed CT” version) in the “Modifications to the data imputation algorithm” subsection of the Methods, in which we stated:*

*“The main disadvantage of this alternative method is that it requires sufficient individuals with complete data, and thus the training data set can be much smaller, resulting in less accurate imputation. The advantage comes when there is a large amount of missing data for some variables, and so we avoid weakening the strength of the edges connected to these variables in the training data set, assuming enough complete data is available”.*

*In terms of the actual *performance in simulations*, this was covered several places within the Results section where we stated:*

*“Fig 1 illustrates that the Imputed CT algorithm is generally effective… The default imputation algorithm does not perform well when only one variable is missing, simulation scenarios (a)-(d), but performs better when the missing data is spread between different variables as seen in simulation scenarios (e) and (f); our default imputation method is really designed for larger networks ideally with sparser missing data. In larger networks, when missing data is replaced with random data (which forms the first step of our default imputation algorithm), we can obtain a fairly accurate initial BN, which leads to better overall performance, as demonstrated below.”*

*“Fig 2 shows…* *Our default imputation method shows overall the highest recall and precision, considerably outperforming using the reduced data set and also outperforming the Imputed CT algorithm.”*

*“We also applied the same methods to one continuous and two discrete data simulation networks taken from the bnlearn BN repository… Our default imputation method shows a clear improvement over using the reduced data set (with the improvement reducing as the proportion of missing data increases), with the Imputed CT algorithm seen to be just slightly less effective.”*

*“Simulation 1…* *There is little difference in performance between our imputation method with or without complete training data.”*

*“Simulation 3…Fig 8 (e) and (f) show an improvement across all BN methods, but in particular when using imputation, especially when complete training data is used.”*

*We have now added some additional discussion regarding the performance of the two imputation methods to the manuscript in relation to the results shown in Figures 3 and 4. We have also added some additional discussion of the relative performance of the two imputation methods, summarising all of the above points, to the Discussion section (lines 442-455).*

*Computational requirements for the two methods were covered in S2 Text, which has now been revised to provide additional details not previously included.*

*The reviewer is correct in stating that we cannot directly compare the performance of BN and CIT in real data, since we do not have any ground truth knowledge. The real data example was supposed to be purely illustrative, but we think it important (particularly for a journal with an applied focus like PLOS Genetics) to show the method at work in practice, in application to real data. We have added a sentence (lines 344-347) pointing out that, as this is a real data set, the truth (or otherwise) of any identified relationships is obviously unknown, and reiterating the fact that further work will be required to confirm (or not) the truth of the potential causal relationships identified by CIT and/or BN.*

Finally, the manuscript is in need of some rewrites, to better cover the area of Bayesian networks generally and BayesNetty specifically in the introduction, and to conform to a manuscript structure that puts Methods at the end.

*We had covered* *Bayesian networks in some detail in our previous paper (Howey et al. 2020: https://pubmed.ncbi.nlm.nih.gov/32119656/) and so we had not considered it necessary to repeat this in the current manuscript. However, as requested, we have now included an even more detailed discussion (!) within the Introduction of the current manuscript.*

*In terms of* *conforming to a manuscript structure that puts Methods at the end, within the Results section we had attempted to point the reader to which sections of the Methods (or Supplementary Text) are most relevant to the Results being described. We have now added some further pointers in this regard.*

* Major comments *

Fig 1 caption: “The title of each plot shows the simulating model and the asterisk denotes which variable or variables are missing in 1800 missing individuals from a total of 2000 individuals (only one variable is missing for each individual).”
Comment: This is quite a significant amount of missingness (90%) and a large number of samples compared to the real data (141 individuals, roughly 25-50% missingness based on the description in lines 168-169) and to the Bayesian Network Repository datasets (500 individuals, approximately 10-40% missing). The large sample size in this 3 variable simulation might help explain why the Reduced method seems to do so well here. Including a simulation study with numbers more similar to that of the real dataset would be an improvement. If not I think justification is needed of why larger sample numbers are used for the small graph simulations.

*We have now added in an AUC (ROC curve) analysis, comparing BN and CIT, using computer simulations based on numbers - i.e. sample sizes and missing data patterns - that are more similar to the real early arthritis data. See the end of Results section and S8 Fig.*

Lines 224-228: “Overall, both the T cell and B cell analyses illustrate a reasonably high correlation between the results from CIT and BN analysis when applied to exactly the same data (Fig 6 (c) and Fig 7 (c)) but less correlation when our imputation approach is used to fill in the missing data, particularly when using a large average network. Further work will be required to confirm (or not) the truth of the causal relationships identified by CIT.”
Comment: It’s unclear what value this comparison (of the results from CIT and from BN analysis) adds.

*We think it important (particularly for a journal with an applied focus like PLOS Genetics) to show the method at work in practice, in application to real data. It therefore seems to us very natural, as part of this illustrative example, to compare our results to those originally obtained using the CIT.*

The layout of the paper is difficult, with results on the performance of the method preceding description of the method itself. Some understanding of the method is needed by the reader to have some intuition when reviewing these results. A first section of the results giving an overview of the method would be useful.

*We agree that this is a difficult structure, with results on the performance of the method preceding description of the method itself, but unfortunately that is the structure that is preferred by PLOS Genetics (and is used in the vast majority of papers published in this journal).*

*In the Results section, we had already attempted to point the reader to which sections of the Methods (or Supplementary Text) are most relevant to the Results being described, and we have now added some further pointers in this regard. It does not seem to us a good use of a limited word limit to repeat large sections of the Methods (or Supplementary Text) within the Results section; we would hope that the interested reader would prove capable of reading the relevant Methods section prior to (or alongside) the relevant Results section if they wish to understand the methodology in more detail. Our new detailed discussion of Bayesian networks within the Introduction of the current manuscript should also prove helpful in terms of understanding the main concepts.*

I also think a more complete review of imputation in Bayesian networks would be useful. For example, Bayesian networks themselves have been used often as a method for imputation, and different imputation methods when fitting Bayesian methods have been considered (eg BCPA, LLS).

*We had already mentioned some previous use of Bayesian networks as a method for imputation in the last paragraph of the Introduction section. We have now expanded the text in this section, and explained how our method relates to these previous methods.*

*The use of different imputation methods when fitting *Bayesian* methods seems to us to not be particularly relevant to our own (*non-Bayesian*) method, and we were unable to find any references to the methods (BCPA, LLS) mentioned by the reviewer. We have therefore chosen not to include anything further on this.*

BayesNetty is not described in any detail so that I can understand what it does, how it does that, which is important as this imputation is built upon it.

*The method implemented in BayesNetty was described in detail both in our original paper (Howey et al. 2020:* [*https://pubmed.ncbi.nlm.nih.gov/32119656/*](https://pubmed.ncbi.nlm.nih.gov/32119656/)*) - which was clearly referenced in the current manuscript - and in the Methods section of the current manuscript (within the subsections “BayesNetty software” and “Average networks”). In addition, as mentioned in the Methods section of the current manuscript, there is documentation (with working examples) freely available on the BayesNetty website http://www.staff.ncl.ac.uk/richard.howey/bayesnetty/*

*As was mentioned in the Methods section of the current manuscript, BayesNetty primarily implements the algorithm implemented in the R package bnlearn, for which full details can be found in the two references provided (Scutari M, Denis JB. Bayesian Networks with Examples in R; Nagarajan R, Scutari M, L ́ebre S. Bayesian Networks in R: with Applications in Systems Biology).*

*We have now provided a much more detailed overview of BNs in general within the Introduction of the current manuscript. We feel that this addition, together with the description given in the Methods section as detailed above, should provide a sufficient basis for the reader to understand what BayesNetty does.*

Why use BayesNetty to build upon, other than because it is the authors’ earlier work?

*The *****practical***** reason to use BayesNetty to build upon is indeed because it is our own earlier work - and thus we have an up and running software package efficiently programmed up in C++ that can be (relatively easily) built upon. The *****scientific***** reason to use BayesNetty to build upon is because it has already been shown in our original paper (Howey et al. 2020:* [*https://pubmed.ncbi.nlm.nih.gov/32119656/*](https://pubmed.ncbi.nlm.nih.gov/32119656/)*) - which was clearly referenced in the current manuscript - to perform competitively with other causal inference methods and, in some cases, to even have advantages over competing approaches, as had already been mentioned in the first paragraph of the Introduction section.*

There is limited information on running time. How long does each run with the real data example take? How does running time scale with the number of individuals/variables? The imputation step alone, which must be run once per individual with missing data, seems intensive.

*We have provided some indication of the scaling with the number of individuals (between 2000 and 12800) and variables (between 31 and 496) in S2 Text, which has been revised to provide additional details not previously included.*

Two imputation approaches are proposed, which seem to beat each other in different circumstances. Could the authors give some intuition about why this is, and which method is more appropriate for different datasets?

*In terms of which of the two imputation methods are optimal in which situations, please see our detailed response, provided further above, to your original question about this.*

*We have now added additional discussion regarding the performance of the two imputation methods to the manuscript in relation to the results shown in Figures 3 and 4. We have also added some additional discussion of the relative performance of the two imputation methods, summarising the overall results, to the Discussion section (lines 442-455).*

Further, there is no exploration about the optimal parameters for imputation. In the either imputation method, should the fraction of the data used for generating the nearest neighbours vary with sample size? What is the advantage of using 90% of the data each time, rather than all the data? Would replacing missing values with the mean for each variable rather than a random value change things?

*We have now explored the use of different fractions of the data in a two new supplementary documents (S3 Text and S9 Fig), which we point to in the main text (lines 444 and 582-583) but we would still recommend using 90% as default. The point of using a subsample (rather than all the data) is to generate some randomness, so that we do not use exactly the same (imperfect)* *best fit network structure to inform the imputation for every individual, but rather resample a new set of training data - and generate a new best fit network structure - before imputing the missing data for each individual. This was already mentioned in the 3rd paragraph of the “Data imputation” section of the Methods (lines 584-596):*

*“To avoid using the same best fit network structure to inform the imputation for every individual, we resample a new set of training data before imputing the missing data for each individual that has missing data. This provides a way to incorporate model uncertainty in the best fit network that is used to inform the imputation.”*

*Replacing missing values with the mean for each variable, rather than a random value, might be an interesting topic for future investigation, but we feel it is beyond the scope of the current (already quite extensive!) manuscript. Similarly, allowing the fraction of the data used for generating the nearest neighbours to vary with sample size would be an interesting topic for future investigation, but we feel it is beyond the scope of the current manuscript.*

The method isn’t universally optimal, particularly with small datasets: e.g. in Figure 1 the authors show that imputation is comparable with EM, and is sometimes worse than using only 10% of the dataset, but that is for just 3 variables. In Fig 4 and Fig 8a-d, they show that imputation is comparable with random initialisation of variables. In Fig 8e-f the imputation methods do outperform random and reduced, though it’s a shame not to see EM in this figure. They claim their method is better on larger networks, so possibly they should include more datasets where their method excels (or e.g. move fig s2 to main, since it actually shows their method narrowly beating EM and random).

*As mentioned further above, it is not possible to include EM in Figure 8, as it does not deal with mixed (discrete and continuous) data.*

*As mentioned in several responses further above, we have now added additional discussion regarding the performance of the two imputation methods to the manuscript in relation to the results shown in Figures 3 and 4. We have also added some additional discussion of the relative performance of the two imputation methods, summarising the overall results, to the Discussion section (lines 442-455).*

It’s unclear how the soft constraints section fits into the rest of the paper, which focuses on imputation. The largest dataset used for analysis of soft constraints had only ~20 nodes, which is small compared to many simulations used in the paper. What link does the soft constraints method have to the imputation method?

*There is no direct link between the* *soft constraints method and the imputation method. Both methods can be considered as improvements and extensions to our original BayesNetty software package, both of which can allow improved detection of causal relationships through improved identification of the best fit network (or networks).*

It’s difficult to understand what is learned by comparing the results on the real datasets obtained by this method relative to an existing method, especially when the outputs of the methods are different (Bayesian estimates vs frequentist p-values). The output of the existing method (CIT) cannot be treated as a ground truth.

*This example was supposed to be purely illustrative, but as further mentioned above, we think it important (particularly for a journal with an applied focus like PLOS Genetics) to show the method at work in practice, in application to real data. We have added a sentence (lines 344-347) pointing out that, as this is a real data set, the truth (or otherwise) of any identified relationships is obviously unknown, and reiterating the fact that further work will be required to confirm (or not) the truth of the potential causal relationships identified by CIT and/or BN.*

* Minor comments *

How many repeats were run for each simulation?

*This was mentioned within the supplementary document S1 Text. Within the “Data imputation simulation with three variables” section we stated “Data were simulated 1000 times to compute powers and type I errors”. Within the “Data imputation simulation with five variables” section we stated: “the whole process was repeated for 1000 simulation replicates”. Within the “Data imputation simulation with examples from the Bayesian Network Repository” section we stated: “The average recall and precision was calculated for the best fit BN using 25 iterations for each method and percentage of missing data”. Within the “Data imputation simulation with many variables” section we stated: “The recall and precision were calculated for each value of beta and averaged over 10 simulation replicates”. Within the “Soft constraint simulations” section we stated: “we used 1000 simulation replicates for each value of beta_A”.*

Line 61 - “Fig 1 shows the power (or Type I error when β=0)”
Comment: I’m unclear on the need or relevance of a hypothesis testing framework here, and am concerned that, as it stands, the bracketed clause reads as an (incorrect) redefinition of the statistical term power. Either the null and alternative hypotheses should be clearly stated, the authors should use the same recall/precision used for future simulation models, or use e.g. “Proportion of runs where best fit network matches”.

*We have now changed the power/type 1 error terminology to “proportion of best fit networks that correctly identified…” and we have made similar changes to S1 Text, the Figure 1 legend, and the labelling of the y axes in Figure 1.*

Line 121 - “We start with a simple model to demonstrate the effect of changing the prior probability of an edge from X to Y”
Comment: Add clarification that the prior is conditional on the edge existing i.e. it is a prior on direction. E.g. “A prior probability is assigned to the direction of an edge between X and Y, conditional on the event that the edge exists.”

*We have now updated this sentence and the description in the “Soft Constraints” section of the Methods to improve clarity. The statement “a prior probability can be assigned to the direction of an edge, conditional on the event that it exists” in the Discussion has also been updated.*

Line 125 - “Provided βA is non-zero, increasing the prior probability of an edge from X to Y increases the power for its detection when it exists (S3 Fig (a)), while only increasing the chance of a false positive detection (S3 Fig (b)) when the directed edge is from Y to X.”
Comment: I found this difficult to understand, particularly from “only increasing the chance…” onwards. Fig S3a seems to have points in the bottom left quadrant, corresponding to when the prior favours Y->X and the best fit networks favour Y->X, which are false positives as in Fig S3a the true model has X->Y. In Fig S3b, there are points in the top right quadrant, corresponding to when the prior favours X->Y and the best fit networks favour X->Y, which again are false positives. So it seems to show that, whether the edge is really X->Y or Y->X there are false positives, though there are few false positives for βA >= 0.4.

*Yes, that is correct, essentially the higher the prior probability from X->Y and the greater the support in the data to correctly orientate the edge between X and Y then the fewer false positives.*

*In S3 Fig (a) the prior favours X->Y when it is greater than 0.5 and favours Y->X when it is less than 0.5. Since the generating model has X->Y, all detections in S3 Fig (a) are true positives.*

*Similarly, in S3 Fig (b) the prior favours X->Y when it is greater than 0.5 and favours Y->X when it is less than 0.5. Since the generating model has Y->X, all detections in S3 Fig (b) are false positives.*

*We have made some edits to the relevant paragraph in the main text to help clarify the interpretation of S3 Fig. We have also made some edits to “Soft Constraints” section of the Methods to improve clarity.*

Line 169 - “Of these 141 individuals, only 68 had no missing data; the other 73 typically only had data for either CD4+ T cells or B cells.”
Comment: Could the authors add information about percentage of values missing, to allow easy comparison with the simulated datasets used?

*Yes, we have now added the overall missing data percentage, 16.9%.*

Line 445 - “We also define an alternative version of our imputation method by making a change to the first step. Specifically we take a 90% subset from the individuals that have complete data (and we thus do not need to randomly replace any missing values). We refer to this alternative method as “imputation with complete training data” and denote it by “imputed CT” in figures.”
Comment: This method is not consistently included in the analysis and not discussed in the discussion.

*We have now consistently included this method in the analysis (specifically, it has been added to Figures 3 and 4), and we have added some discussion of it to the Discussion section (lines 442-455).*

Fig 3
Comment: The grey lines (edges missing from the graph), as the authors note, are “almost invisible”. Perhaps the grey lines could be made darker, and the orange lines more distinct from the red. A legend would be very helpful. Could a network for Random be included?

*We have now made the grey lines darker, and have added a network for Random as well as for “imputed CT”. It was hard to find a way to include a legend on the plot itself, but full details are provided within the figure legend.*

Fig 4
Comment: Is there a reason for Imputed-CT and EM to be excluded from this graph? In general it would be helpful for comparison if all methods were included in all plots (e.g. EM is also excluded from Fig 8)

*We have now included “imputed CT” in Figure 4. It is not possible to include EM in Figures 4 and 8, as EM does not work with mixed (discrete and continuous) data. We have added a note to this effect in the relevant places in the manuscript main text.*

Figs 6, 7
Comment: What do the different colours and shapes for the points mean? Are they necessary?

*We have now added a description of what the different colours and shapes for the points mean to the Figure legends.*

Text S2: Computational feasibility and timings.
Comment: It would be useful to be able to compare timings between the methods used in the paper i.e. Imputed, Imputed-CT, Reduced, Full and EM. It’s useful to be able to see how the timings scale with the number of variables (nodes), but it would also be useful to see how it scales with the number of samples. In the tables in S2, I would suggest changing the label of “Simple Parallel” to “Parallel (1000)” to make it clear it requires 1000 parallel processes.

*We have now added a table in Text S2 showing how the timings scale with the number of samples (individuals). We have also added a table comparing timings between our imputation approaches and the EM algorithm. We have also changed the column heading “Simple Parallel” to “Parallel (1000)”, as suggested.*

**Have all data underlying the figures and results presented in the manuscript been provided?**
Large-scale datasets should be made available via a public repository as described in the *PLOS Genetics* [data availability policy](https://eur03.safelinks.protection.outlook.com/?url=http%3A%2F%2Fjournals.plos.org%2Fplosgenetics%2Fs%2Fdata-availability&data=04%7C01%7Cheather.cordell%40newcastle.ac.uk%7Cee828810aa184543ea0908d8e8be5bf6%7C9c5012c9b61644c2a91766814fbe3e87%7C1%7C0%7C637515252610258155%7CUnknown%7CTWFpbGZsb3d8eyJWIjoiMC4wLjAwMDAiLCJQIjoiV2luMzIiLCJBTiI6Ik1haWwiLCJXVCI6Mn0%3D%7C3000&sdata=FnSxKCBrh7ULlvAsFwNPfImiXxnA0CNzPEoT4Pjh6Gg%3D&reserved=0), and numerical data that underlies graphs or summary statistics should be provided in spreadsheet form as supporting information.

Reviewer #1: Yes

Reviewer #2: Yes

Reviewer #3: **No:** Could not find scripts and data used for simulations. Not clear which scripts in https://github.com/aclark5/Lymphocyte_meQTL relate to this paper. Annotation could be improved (eg - give a hint in the README which scripts to look at!)

*We have now amended the data availability statement to clarify where the data and scripts can be found. Specifically, it now reads:*

*"All relevant simulated data are within the manuscript and its Supporting Information files. Scripts underlying the computer simulations and the BN and CIT analysis of the processed DNA methylation and gene expression data from Clark et al. (2020) may be found at* [*https://github.com/drhowey/BayesNetty/blob/main/docs/scripts.zip*](https://eur03.safelinks.protection.outlook.com/?url=https%3A%2F%2Fgithub.com%2Fdrhowey%2FBayesNetty%2Fblob%2Fmain%2Fdocs%2Fscripts.zip&data=04%7C01%7Cheather.cordell%40newcastle.ac.uk%7C0791adca7bdf45a209aa08d916df9c80%7C9c5012c9b61644c2a91766814fbe3e87%7C1%7C0%7C637565971920010248%7CUnknown%7CTWFpbGZsb3d8eyJWIjoiMC4wLjAwMDAiLCJQIjoiV2luMzIiLCJBTiI6Ik1haWwiLCJXVCI6Mn0%3D%7C1000&sdata=%2FDOl7XEa0rAYSZT30LUpS5wNeEFxnQy21bEf6PPXJXs%3D&reserved=0)*. The original DNA methylation data from CD4 and B cells used in this study, together with paired transcriptome data, are available in the GeneExpression Omnibus database (accession no. GSE137634;* [*http://www.ncbi.nlm.nih.gov/geo*](https://eur03.safelinks.protection.outlook.com/?url=http%3A%2F%2Fwww.ncbi.nlm.nih.gov%2Fgeo&data=04%7C01%7Cheather.cordell%40newcastle.ac.uk%7Cf66406977791412b8e8108d90a7c7606%7C9c5012c9b61644c2a91766814fbe3e87%7C1%7C0%7C637552351934239514%7CUnknown%7CTWFpbGZsb3d8eyJWIjoiMC4wLjAwMDAiLCJQIjoiV2luMzIiLCJBTiI6Ik1haWwiLCJXVCI6Mn0%3D%7C1000&sdata=GMa0%2FUhfa8ZlohDK3BrdQPW1tOuIGrLYFGUMeeGEnx0%3D&reserved=0)*). Annotated scripts used for the processing and CIT analysis of these data as performed by Clark et al. (2020), together with the input file used for the current work, may be found at* [*https://github.com/aclark5/Lymphocyte_meQTL*](https://eur03.safelinks.protection.outlook.com/?url=https%3A%2F%2Fgithub.com%2Faclark5%2FLymphocyte_meQTL&data=04%7C01%7Cheather.cordell%40newcastle.ac.uk%7Cf66406977791412b8e8108d90a7c7606%7C9c5012c9b61644c2a91766814fbe3e87%7C1%7C0%7C637552351934239514%7CUnknown%7CTWFpbGZsb3d8eyJWIjoiMC4wLjAwMDAiLCJQIjoiV2luMzIiLCJBTiI6Ik1haWwiLCJXVCI6Mn0%3D%7C1000&sdata=HhPZSdBaAB6XXGf3bOT2JEWoriVL3kI6VTwRJ4a7BXQ%3D&reserved=0)*. "*

PLOS authors have the option to publish the peer review history of their article ([what does this mean?](https://eur03.safelinks.protection.outlook.com/?url=https%3A%2F%2Fjournals.plos.org%2Fplosgenetics%2Fs%2Feditorial-and-peer-review-process%23loc-peer-review-history&data=04%7C01%7Cheather.cordell%40newcastle.ac.uk%7Cee828810aa184543ea0908d8e8be5bf6%7C9c5012c9b61644c2a91766814fbe3e87%7C1%7C0%7C637515252610268115%7CUnknown%7CTWFpbGZsb3d8eyJWIjoiMC4wLjAwMDAiLCJQIjoiV2luMzIiLCJBTiI6Ik1haWwiLCJXVCI6Mn0%3D%7C3000&sdata=MGFcGqdvW%2FifYyTr42Z9Qtf06OrOBREKnFLEjcLfIF4%3D&reserved=0)). If published, this will include your full peer review and any attached files.


If you choose “no”, your identity will remain anonymous but your review may still be made public.


**Do you want your identity to be public for this peer review?** For information about this choice, including consent withdrawal, please see our [Privacy Policy](https://eur03.safelinks.protection.outlook.com/?url=https%3A%2F%2Fwww.plos.org%2Fprivacy-policy&data=04%7C01%7Cheather.cordell%40newcastle.ac.uk%7Cee828810aa184543ea0908d8e8be5bf6%7C9c5012c9b61644c2a91766814fbe3e87%7C1%7C0%7C637515252610268115%7CUnknown%7CTWFpbGZsb3d8eyJWIjoiMC4wLjAwMDAiLCJQIjoiV2luMzIiLCJBTiI6Ik1haWwiLCJXVCI6Mn0%3D%7C3000&sdata=K4E9nw3xHOP1Bs%2F6JULHxEzWmvKYzXY4f7eh7SP1IZw%3D&reserved=0).

Reviewer #1: No

Reviewer #2: **Yes:** Jack Bowden

Reviewer #3: No
