# Supplementary material for: A Bayesian network approach incorporating imputation of missing data enables exploratory analysis of complex causal biological relationships
Source: PLoS Genet. 2021 Sep 29;17(9):e1009811. doi: 10.1371/journal.pgen.1009811 (PMC8504979; doi:10.1371/journal.pgen.1009811)
Supplement: Attachment — Submitted filename: ResponseToSecondReview.docx [file pgen.1009811.s014.docx]

Dear Dr Cordell,

Thank you very much for submitting your Research Article entitled 'A Bayesian network approach incorporating imputation of missing data enables exploratory analysis of complex causal biological relationships' to PLOS Genetics.

The manuscript was fully evaluated at the editorial level and by independent peer reviewers. The reviewers appreciated the attention to an important topic but identified some concerns that we ask you address in a revised manuscript.

We find that the authors have addressed most of the comments that the reviewers made on the previous submission. However, Reviewer #1 feels that some of the points he made in his review have not been addressed in full. In particular, we agree that the paper could be made more compact by dropping material that is less central; and that any apparent issues with data double-dipping should be clarified. Please also consider at least one other method of imputation to compare with the proposed method.

*We thank the editor for their suggestions. Although (as we note in our comments to the reviewer below) the main aim of our manuscript was NOT to develop a new method of imputation per se, we have nevertheless now added in a comparison with three existing imputation methods, as requested.*

*We are still unable to understand the reviewer’s concerns over “data double-dipping”, however, to avoid getting into further discussion of this aspect, we have chosen to remove this section. This also has the advantage of making the paper more compact.*

*We have removed some other less central material in an effort to make the paper more compact, but we are quite reluctant to remove the things specifically requested by the reviewer (namely (1) everything related to “soft constraints”, (2) all the CIT vs BayesNetty comparisons, (3) every mention of “causality” and related text) for the following reasons:*

(1) *Given that, as mentioned below, the aim of our manuscript was to improve the performance of a standard (previously-developed) BN method through* ***two*** *improvements, (a) the incorporation of a semi-novel imputation approach and (b) the incorporation of soft constraints, it seems to us a bit odd to remove everything related to “soft constraints”.*
(2) *Given that we have submitted our manuscript to a genetics journal, rather than a statistics journal, it seems to us that the applied example will be of interest to the readers of PLOS Genetics. And, since the applied example applies our approach to data that was previously analysed using CIT, it seems to us not unreasonable to include comparisons of CIT vs BayesNetty.*
(3) *Given that we were specifically asked at the last round of reviews by several reviewers to address the issue of causality (and the extent to which the edges in a BN could be considered to represent causal relationships), we consider this is important material that should probably be retained.*

*We consider at this point that it is really an editorial decision about whether any of the material listed above needs to be removed. We imagine that the reviewer will not be satisfied with our response (!) but we would respectfully point out that two other reviewers have already recommended acceptance of the manuscript without requiring removal of this material.*

We therefore ask you to modify the manuscript according to the review recommendations. Your revisions should address the specific points made by each reviewer.

In addition we ask that you:
1) Provide a detailed list of your responses to the review comments and a description of the changes you have made in the manuscript.
2) Upload a Striking Image with a corresponding caption to accompany your manuscript if one is available (either a new image or an existing one from within your manuscript). If this image is judged to be suitable, it may be featured on our website. Images should ideally be high resolution, eye-catching, single panel square images. For examples, please browse our [archive](https://eur03.safelinks.protection.outlook.com/?url=https%3A%2F%2Fjournals.plos.org%2Fplosgenetics%2Fvolume&data=04%7C01%7Cheather.cordell%40newcastle.ac.uk%7C7a83023f5e51434d08d908d93d2e0d15%7C9c5012c9b61644c2a91766814fbe3e87%7C1%7C0%7C637608090262659519%7CUnknown%7CTWFpbGZsb3d8eyJWIjoiMC4wLjAwMDAiLCJQIjoiV2luMzIiLCJBTiI6Ik1haWwiLCJXVCI6Mn0%3D%7C1000&sdata=YUlnc2Uhs4oE30pd4dQ2BLQLYt4diE2M9rScrg6kW6o%3D&reserved=0). If your image is from someone other than yourself, please ensure that the artist has read and agreed to the terms and conditions of the Creative Commons Attribution License. Note: we cannot publish copyrighted images.

We hope to receive your revised manuscript within the next 30 days. If you anticipate any delay in its return, we would ask you to let us know the expected resubmission date by email to plosgenetics@plos.org.

If present, accompanying reviewer attachments should be included with this email; please notify the journal office if any appear to be missing. They will also be available for download from the link below. You can use this link to log into the system when you are ready to submit a revised version, having first consulted our [Submission Checklist](https://eur03.safelinks.protection.outlook.com/?url=http%3A%2F%2Fjournals.plos.org%2Fplosgenetics%2Fs%2Fsubmit-now%23loc-submission-checklist&data=04%7C01%7Cheather.cordell%40newcastle.ac.uk%7C7a83023f5e51434d08d908d93d2e0d15%7C9c5012c9b61644c2a91766814fbe3e87%7C1%7C0%7C637608090262659519%7CUnknown%7CTWFpbGZsb3d8eyJWIjoiMC4wLjAwMDAiLCJQIjoiV2luMzIiLCJBTiI6Ik1haWwiLCJXVCI6Mn0%3D%7C1000&sdata=XTdkPKx4BtlTIKxccxIu4qfVTuzl336Z29C3buUgWAw%3D&reserved=0).

While revising your submission, please upload your figure files to the [Preflight Analysis and Conversion Engine](https://eur03.safelinks.protection.outlook.com/?url=http%3A%2F%2Fpace.apexcovantage.com%2F&data=04%7C01%7Cheather.cordell%40newcastle.ac.uk%7C7a83023f5e51434d08d908d93d2e0d15%7C9c5012c9b61644c2a91766814fbe3e87%7C1%7C0%7C637608090262669474%7CUnknown%7CTWFpbGZsb3d8eyJWIjoiMC4wLjAwMDAiLCJQIjoiV2luMzIiLCJBTiI6Ik1haWwiLCJXVCI6Mn0%3D%7C1000&sdata=kTW9Oesrz13ZhNmMt5e%2BEBhTfuQxRDm7bsaXtz2nukI%3D&reserved=0) (PACE) digital diagnostic tool. PACE helps ensure that figures meet PLOS requirements. To use PACE, you must first register as a user. Then, login and navigate to the UPLOAD tab, where you will find detailed instructions on how to use the tool. If you encounter any issues or have any questions when using PACE, please email us at figures@plos.org.

Please be aware that our [data availability policy](https://eur03.safelinks.protection.outlook.com/?url=http%3A%2F%2Fjournals.plos.org%2Fplosgenetics%2Fs%2Fdata-availability&data=04%7C01%7Cheather.cordell%40newcastle.ac.uk%7C7a83023f5e51434d08d908d93d2e0d15%7C9c5012c9b61644c2a91766814fbe3e87%7C1%7C0%7C637608090262669474%7CUnknown%7CTWFpbGZsb3d8eyJWIjoiMC4wLjAwMDAiLCJQIjoiV2luMzIiLCJBTiI6Ik1haWwiLCJXVCI6Mn0%3D%7C1000&sdata=ftESjbMNwkJjOHlKqOe60RuCtweN8g5tULMYJPP%2FOYc%3D&reserved=0) requires that all numerical data underlying graphs or summary statistics are included with the submission, and you will need to provide this upon resubmission if not already present. In addition, we do not permit the inclusion of phrases such as "data not shown" or "unpublished results" in manuscripts. All points should be backed up by data provided with the submission.

To enhance the reproducibility of your results, we recommend that you deposit your laboratory protocols in protocols.io, where a protocol can be assigned its own identifier (DOI) such that it can be cited independently in the future. Additionally, PLOS ONE offers an option to publish peer-reviewed clinical study protocols. Read more information on sharing protocols at [https://plos.org/protocols?utm_medium=editorial-email&utm_source=authorletters&utm_campaign=protocols](https://eur03.safelinks.protection.outlook.com/?url=https%3A%2F%2Fplos.org%2Fprotocols%3Futm_medium%3Deditorial-email%26utm_source%3Dauthorletters%26utm_campaign%3Dprotocols&data=04%7C01%7Cheather.cordell%40newcastle.ac.uk%7C7a83023f5e51434d08d908d93d2e0d15%7C9c5012c9b61644c2a91766814fbe3e87%7C1%7C0%7C637608090262679428%7CUnknown%7CTWFpbGZsb3d8eyJWIjoiMC4wLjAwMDAiLCJQIjoiV2luMzIiLCJBTiI6Ik1haWwiLCJXVCI6Mn0%3D%7C1000&sdata=crB6isuufvB%2FH7qXMLVfBT20eGVwVjuASF1lberSG54%3D&reserved=0)

Please review your reference list to ensure that it is complete and correct. If you have cited papers that have been retracted, please include the rationale for doing so in the manuscript text, or remove these references and replace them with relevant current references. Any changes to the reference list should be mentioned in the rebuttal letter that accompanies your revised manuscript. If you need to cite a retracted article, indicate the article’s retracted status in the References list and also include a citation and full reference for the retraction notice.

PLOS has incorporated [Similarity Check](https://eur03.safelinks.protection.outlook.com/?url=http%3A%2F%2Fwww.crossref.org%2Fcrosscheck.html&data=04%7C01%7Cheather.cordell%40newcastle.ac.uk%7C7a83023f5e51434d08d908d93d2e0d15%7C9c5012c9b61644c2a91766814fbe3e87%7C1%7C0%7C637608090262679428%7CUnknown%7CTWFpbGZsb3d8eyJWIjoiMC4wLjAwMDAiLCJQIjoiV2luMzIiLCJBTiI6Ik1haWwiLCJXVCI6Mn0%3D%7C1000&sdata=WEkuq4dLRrnIGgAgiJUarp%2BzDNnShGIgwN3LoCDMumA%3D&reserved=0), powered by iThenticate, into its journal-wide submission system in order to screen submitted content for originality before publication. Each PLOS journal undertakes screening on a proportion of submitted articles. You will be contacted if needed following the screening process.

To resubmit, you will need to go to the link below and 'Revise Submission' in the 'Submissions Needing Revision' folder.


Please let us know if you have any questions while making these revisions.

Yours sincerely,

Marco Scutari
Guest Editor
PLOS Genetics

David Balding
Section Editor: Methods
PLOS Genetics


Reviewer's Responses to Questions

**Comments to the Authors:**

Reviewer #1: Well, I feel I understand this paper better now, although certainly it is not a complete understanding. Much of the Bayesian network discussion and material is actually tangential to the main purpose.

*Yes, we agree that much of the Bayesian network discussion and material is actually tangential to the main purpose. However, we were specifically asked at the last round of reviews by several reviewers (including yourself) to include this material, in order to help set the scene (and perhaps enable the reader to gain a better understanding of the methodology).*

As I understand it, the authors are presenting a new method of imputation specifically just for BN edge reconstruction (and specifically just for use with their own BN method, BayesNetty), which is a combination of preliminary BN structure learning and nearest-neighbor imputation, and therefore they only test their imputation by the quality of BN edge reconstruction the imputation enables.
First, if they’re going to propose new methods of imputation, they should be tested against existing methods of imputation. This is mostly not done. There’s no testing against imputing by the mean, hotdecking, regression based, existing nearest-neighbor methods, or other probabilistic imputation, except for EM, which they then fail to use in many of the cases considered (they claim that EM is unsuitable to mixed networks, but the basic idea of EM is very simple and very general, I find this argument lacking merit. However, even a straightforward extension of EM to the hybrid continuous-discrete network case would be more appropriate to a separate paper).

*The reviewer has misunderstood the purpose of our manuscript. Our purpose is NOT to propose a new method of imputation. It is rather to improve the performance of a standard (previously-developed) BN method (the score-based algorithm proposed by Scutari and Dennis (2014) as implemented by Marco Scutari in the R package bnlearn)* ***through two new extensions****, namely (a) the incorporation of a semi-novel imputation approach and (b) the incorporation of soft constraints. We have added some sentences to the Abstract and Lay summary to clarify this point.*

*We feel this purpose is already well-reflected in the first part of the title “A Bayesian network approach incorporating imputation of missing data enables…” which clearly indicates that the focus of the manuscript is on* ***the BN method****, and the extent to which it can be* ***improved*** ***through incorporation*** *of imputation.*

*The reviewer is correct in that by “performance” we mean the quality of the BN edge reconstruction. We feel that, to the extent to which directed edges can be considered to represent potential causal biological relationships between variables (see discussion later), this purpose is already well-reflected in the second part of the title “…enables exploratory analysis of complex causal biological relationships”.*

*Had our purpose been to propose a new method of imputation, we would have chosen a different title such as (for example) “A new method of missing data imputation using Bayesian Networks”.*

Second, if they’re going to propose methods of imputation for edge reconstruction, they should test imputation with multiple methods of BN edge reconstruction, instead of just their own software BayesNetty. It seems like hillclimbing, K2, basically any algorithm, with or without backtracking and restarts, could be used for either the BN-aided imputation portion of the algorithm, or for the BN edge reconstruction portion of this endeavor that serves as the metric.

*We feel that it is beyond the scope of the current manuscript to evaluate multiple different methods for edge reconstruction. We already acknowledge in the introduction that there are a variety of different algorithms that have been proposed to search for causal models and/or BNs that are well-supported by a given set of data, and we provide references to them.*

*The reviewer is correct that one could use alternative methods for the BN edge reconstruction portion of the endeavour (even if one had first used BayesNetty for the BN-aided imputation step) and so it would be quite easy for any researcher who wishes to investigate this aspect to explore it themselves (using BayesNetty for the imputation step, and whatever method/software they prefer for the BN edge reconstruction step). Given that the reviewer already considers our paper to be too long (and suggests splitting it over two or more papers, see below) it would seem beyond the scope of the current manuscript to add in this whole additional line of investigation.*

Third, the idea that the best metric of imputation-quality is BN edge reconstruction requires some justification. What is the difference between using this metric and the metric of just “accuracy of imputation”? A comparison could easily be presented using the existing simulation studies.

*As mentioned above (and several times in the manuscript), our purpose is NOT to generate accurate imputation of missing data, but rather to improve the identification of the best fit network (or networks). Therefore, the appropriate metric to use for this purpose is NOT imputation accuracy but rather the accuracy of the network (or edge) reconstruction.*

If they could fix the first problem, this work would be of interest to a limited group: people using BayesNetty for edge reconstruction in datasets with missing data, who decide that imputation is their best option. A re-titling would help make the work more clear:
“BN-assisted nearest-neighbor imputation of missing data to optimize edge reconstruction of BayesNetty software”

*As mentioned above, we feel that our primary purpose is already well-reflected both in the second part of the current title “…enables exploratory analysis of complex causal biological relationships” - which clearly indicates that the main goal is to optimize network reconstruction - and in the first part of the current title “A Bayesian network approach incorporating imputation of missing data enables…” - which clearly indicates that the focus of the manuscript is on the BN method, and the extent to which it can be improved through incorporation of imputation.*

*The reviewer is correct that one could use alternative methods for the BN edge reconstruction portion of the endeavour (even if one had first used BayesNetty for the BN-aided imputation step). Thus it would be a bit misleading to make “edge reconstruction of BayesNetty software” the main focus of the title - even though it is true that we ourselves, for convenience, use our own BayesNetty software implementation (of the Scutari and Dennis score-based algorithm).*

Length / Complexity / Focus:

The paper is now over 30 pages of single-spaced text with another 8 pages of figures, and 12 separate supplemental materials. This should probably be split into two papers (or more), or greatly streamlined.

*We are a bit surprised that the reviewer considers there is a sufficient body of scientific material in our paper for it to be split over two or more papers! We agree that, in responding to the many reviewer comments from the previous review, the manuscript has now become a bit unwieldy, and we have endeavored to condense it a bit. However, we would point out that the number of figures and supplemental materials are not substantially different from (and indeed in many cases are rather fewer than) those of other papers published in PLOS Genetics, for example:*

<https://journals.plos.org/plosgenetics/article?id=10.1371/journal.pgen.1009596>

<https://journals.plos.org/plosgenetics/article?id=10.1371/journal.pgen.1009021>

<https://journals.plos.org/plosgenetics/article?id=10.1371/journal.pgen.1008973>

At a first glance, I would suggest cutting: (1) everything related to “soft constraints”. (2) all the CIT vs BayesNetty comparisons. (3) every mention of “causality” and related text (see below).

*While we understand the reviewer’s desire for us to streamline and condense the manuscript, we would respectfully point out that:*

1. *Given that, as mentioned above, the aim of our manuscript was to improve the performance of a standard (previously-developed) BN method through two improvements, (a) the incorporation of a semi-novel imputation approach and (b) the incorporation of soft constraints, it seems to us a bit odd to remove everything related to “soft constraints”.*
2. *Given that we have submitted our manuscript to a genetics journal, rather than a statistics journal, it seems to us that the applied example will be of interest to the readers of PLOS Genetics. And, since the applied example applies our approach to data that was previously analysed using CIT, it seems to us not unreasonable to include comparisons of CIT vs BayesNetty.*
3. *Given that we were specifically asked at the last round of reviews by several reviewers to address the issue of causality (and the extent to which the edges in a BN could be considered to represent causal relationships), we consider this is important material that should probably be retained.*

Minor new points:
I suggest they provide a name for their newly proposed imputation method. (BN-Assisted 1-Nearest Neighbor Imputation: BNANNI, "Banana"?)

*Given that the main aim of our manuscript was NOT to develop a new method of imputation per se, we are not convinced of the need for an acronym of this type.*

Previous Points:
Causality
I don’t know that the text added by the authors really helped. It could be perhaps condensed. As the authors have described, some people (famously including Pearl himself) consider the arrows of BNs to be “causal” while others observe that there’s nothing in the semantics requiring a causal interpretation. But as this discussion in the authors’ new text shows, if one is given a BN or one generates a BN, one could then assume the edges to be causal based on what comes down to personal preference. However, that assumption could then be true or false, for each edge, as the case may be. It doesn’t seem like calling edges of recreated biological Bayesian networks “causal” really gains anything – if the edges are between entities that are assumed to have a causal relationship based on current biological understanding, then those edges may well represent a causal link. And if not, then calling it “causal” is probably incorrect. Nothing is really gained in either case.

*We are sorry that the reviewer does not consider that the text that we added has helped. We have attempted to add a few further clarifications, as we think this is an important issue. However, we are struggling to fully understand the points that the reviewer is making.*

*It seems to us that the definition of causality that we are using (in terms of the probability distribution of one variable being determined by, or responsive to any change in, the value of another) is not an unreasonable definition. The set of such causal relationships can be encoded as (causal) BN in which directed arcs represent direct functional relationships among the corresponding variables. Comparisons of the fits of different (acausal, probabilistic) networks to the observed data provides a logical way to determine which causal networks (=sets of potential causal relationships) are supported by (or are even compatible with) the observed data and which causal networks (=sets of potential causal relationships) are essentially ruled out by the observed data.*

*Thus, what is gained is the identification of potential causal relationships (and, perhaps more importantly, a ruling out of relationships that, based on the observed data, seem unlikely to be causal).*

Including assumptions (1) (2) and (3) required for causality with no discussion of them is a little strange. For example, (1) isn’t a property of BNs in general. It’s a property of BNs that “accurately depict causality”, which is contingent on the domain being modeled, and a property that requires potentially difficult verification for general biological networks.

*We had never stated that assumption (1) was a property of BNs in general. We rather stated that Spirtes (2010) had stated that (1) was an assumption required in order to use samples from probability densities to make causal inferences.*

*We have attempted to add further clarification to this section. However, given that the reviewer considers that the manuscript is already too long (and indeed suggests removing all references to causality!) it seems to us that it would be beyond the scope of the current manuscript to include any further discussion of these assumptions.*

I would suggest just removing all references to causality in an effort to simplify and streamline this paper.

*Given the importance of this issue, we do NOT consider it a satisfactory solution to just remove all references to causality. Indeed, it seems to us that this would cause even greater confusion! We were specifically asked at the last round of reviews by several reviewers to address the issue of causality (and the extent to which the edges in a BN could be considered to represent causal relationships), and so we consider this as important material that should probably be retained.*

Throwing out whole samples.
It still seems to me that this could be remedied by adjusting equivalent assumed sample sizes using the priors; but I admit there’s no existing theoretical justification for this; placing this issue beyond the scope of the current investigation.
However, it doesn’t seem like throwing out whole samples is what’s typically done. Imputation of some sort seems much more likely to this reviewer. There’s no justification for this statement. Why is there no comparison to just (e.g.) imputing with the mean? Or any of the top 5 or six imputation methods? Most critically, imputation by nearest-neighbor is not new; there must be a comparison to existing nearest neighbor imputation methods.

*We have now included a comparison with several* *existing nearest neighbor imputation methods, specifically (a) imputing using the mean, (b) standard nearest neighbor imputation (using all available variables to determine the nearest neighbor) and (c) multiple imputation using chained equations (MICE) implemented in the mi R package. We note that MICE by definition generates multiple imputed (filled-n) data sets that must be separately used for BN construction. For each simulation replicate, we therefore calculate the average recall and precision (or, for the 3-variable simulation, the proportion of correctly-identified networks) across the multiple imputed data sets, which are then averaged across simulation replicates.*

Data double-dipping: (for evaluation of their imputation method on real datasets)
This still seems like a serious problem that has gone unaddressed. The authors are using their algorithm to perform imputation, then build a network, then generate data with the same missingness patterns as were addressed by imputation, then testing their imputation method against other paradigms based on how well a BN can be recreated based on the (imputed) data. This will systematically overestimate the performance of their imputation.
This system is clever as a method for evaluating accuracy in their framework; it just can’t start and end with using their own algorithm to evaluate their own algorithm’s accuracy.

*We are still unclear why the reviewer feels there is a “data double-dipping” issue. We simulate data from a specific assumed network, we set some of the simulated data to missing, and we then see how well our algorithm (which involves imputing the data via our BN approach then fitting a best-fit network to the resulting “filled-in” data set) performs in comparison to other algorithms, in terms of reconstructing the known edges of the simulation network.*

*The fact that the assumed simulation network was generated through an initial run of imputation followed by network construction on a real data set seems to us irrelevant to the validity or otherwise of the above procedure. Can the reviewer explain why they think this procedure should systematically overestimate the performance of our algorithm?*

*In any case, although we do NOT agree with the reviewer on this point, to avoid getting into further discussion of this aspect we have chosen to remove this section. This also has the advantage of considerably shortening the paper.*

Reviewer #2: I am happy that the reviewers have addressed my comments in the revised version, and the clarity of the paper has improved as a result. I have no further comments on the paper and am happy for it to be accepted

Reviewer #3: I do not see any major errors in this work that prevent publication. Concerns about how the paper is structured which pose a barrier to a reader understanding their work remain, but the authors have chosen not to address these.
